# Foodomics: Analytical Opportunities and Challenges

**DOI:** 10.1021/acs.analchem.1c04678

**Published:** 2021-11-23

**Authors:** Alberto Valdés, Gerardo Álvarez-Rivera, Bárbara Socas-Rodríguez, Miguel Herrero, Elena Ibáñez, Alejandro Cifuentes

**Affiliations:** Laboratory of Foodomics, Institute of Food Science Research, CIAL, CSIC, Nicolas Cabrera 9, Madrid, 28049, Spain

Food analysis has traditionally been an important field of application of analytical chemistry. Indeed, administrations, governments, consumers, and researchers worldwide rely on analytical chemistry to ensure that we all consume safe food products. Although food safety has been the primary goal of food analysis, aspects related to food quality, food traceability, and processing have gained importance progressively. Along this line, the constant evolution of analytical tools in the last decades has allowed researchers to move from classical procedures characterized by targeting a limited number of analytes and modest analytical performance, to advanced methodologies in which the latest advances in the field are applied to food science. This application permits one to pursue more ambitious aims, looking for an increase on the scientific evidence, thanks to the attainment of a broader, more complex, perspective. As a result, research in food science has significantly been benefited and, particularly, studies dealing with the connection between food and health have received a big push, considering the complex relationships that must be assessed under this topic.

In the midst of this evolution in food analysis, the term “Foodomics” was defined to integrate the use of advanced omics technologies, such as transcriptomics, proteomics, and metabolomics, together with biostatistics, chemometrics, and bioinformatics, to allow the evaluation of complex biological systems, as well as the mechanisms of bioactive food compounds that may affect them.^1,2^ The application of these methodologies have permitted a dramatic change in the field of food science, as the research performed may be reoriented to discover new associations in every studied topic. For instance, through Foodomics-related applications, our knowledge regarding the binomial between food and health has been widened. The use of massive omics techniques, such as genomics, transcriptomics, proteomics, metabolomics, nutrigenetics, nutrigenomics, and microbiomics, among others, all of them essential tools employed in Foodomics, will still make it possible to unravel the huge complexity of the Foodome which has been defined as the pool of all compounds present in a food sample and/or in a biological system interacting with the investigated food at a given time.^3^ Consequently, the outputs of this research are expected to be decisive, as diet is one of the most modifiable factors affecting health.

Besides, the availability of instrumentation possessing greater analytical capability has facilitated to change the way in which classical procedures were applied, increasing the attainable performance and reaching new conclusions. As a result, all the subfields including food safety, quality, traceability, and processing, in addition to the study of food and health, have received a great boost. This change is easily observable throughout recent years and, at this point, is where the importance of Foodomics and modern food analysis to analytical chemistry stands. Table 1 shows a summary of the most recent review papers published related to the topic of the present work, highlighting the interest that lies behind the application of Foodomics at present.

**Table 1:** Review Papers on Foodomics Applications Published in the Period Covered by This Work (September 2019–September 2021)

subject	publication year	reference
Foodomics on proteomics studies of beef characterization	2021	(4)
Foodomics on proteomics studies of cross-linking reactions	2021	(5)
Foodomics for understanding protective effect of polyphenols	2021	(6)
proteomics applications in health studies	2021	(7)
Foodomics on functional and activity studies of plant polyphenols	2021	(8)
metabolomics as a tool to study underused soy parts	2021	(9)
capillary electromigration–mass spectrometry in food analysis	2021	(10)
Foodomics for meat quality assessment	2021	(11)
Foodomics studies about bioactive peptides in marine organisms	2021	(12)
Foodborne pathogens evaluation using omics techniques	2021	(13)
Foodomics on food quality and safety assessment	2021	(14)
data mining/machine learning methods in foodomics	2021	(15)
omics and nutrition studies for food characterization	2021	(16)
application of omics in biology system studies	2021	(17)
mass spectrometry-based lipidomics as platform in foodomics research	2021	(18)
chemometrics, 2D-gas chromatography and omics sciences studies	2021	(19)
influence of diet on kidney diseases	2021	(20)
Foodomics on bee product research	2021	(21)
Metabolomics for food safety and food quality studies	2021	(22)
omics in the study of fermented food and beverages	2021	(23)
miniaturized LC in molecular omics	2020	(24)
modeling foodomics data for nutrients bioaccessibility studies	2020	(25)
Foodomics on table olive fermentation studies	2020	(26)
food quality assessed by chemometrics	2020	(27)
microbiological quality of plant-based dietary supplements	2020	(28)
virgin olive oil metabolomics	2020	(29)
2D-liquid chromatography approaches in Foodomics	2019	(30)
advances in research on diabetes by human nutriomics	2019	(31)
organic monolithic capillary columns applications in food analysis	2019	(32)
basic principles and practice of sensomic	2019	(33)
nanoscale separations based on LC and CE for food analysis	2019	(34)

Thus, the present review is intended to cover the most recent advancements on this active field of research during the last two years (September 2019 to September 2021) in a critical way, showing the most important capabilities and possibilities offered by the application of Foodomics strategies, together with the most critical challenges that remain to be solved. Readers interested on more specific groups of applications are referred to the published reviews that are summarized in Table 1.

## Analytical Tools and Opportunities in Foodomics

**Figure 1:**
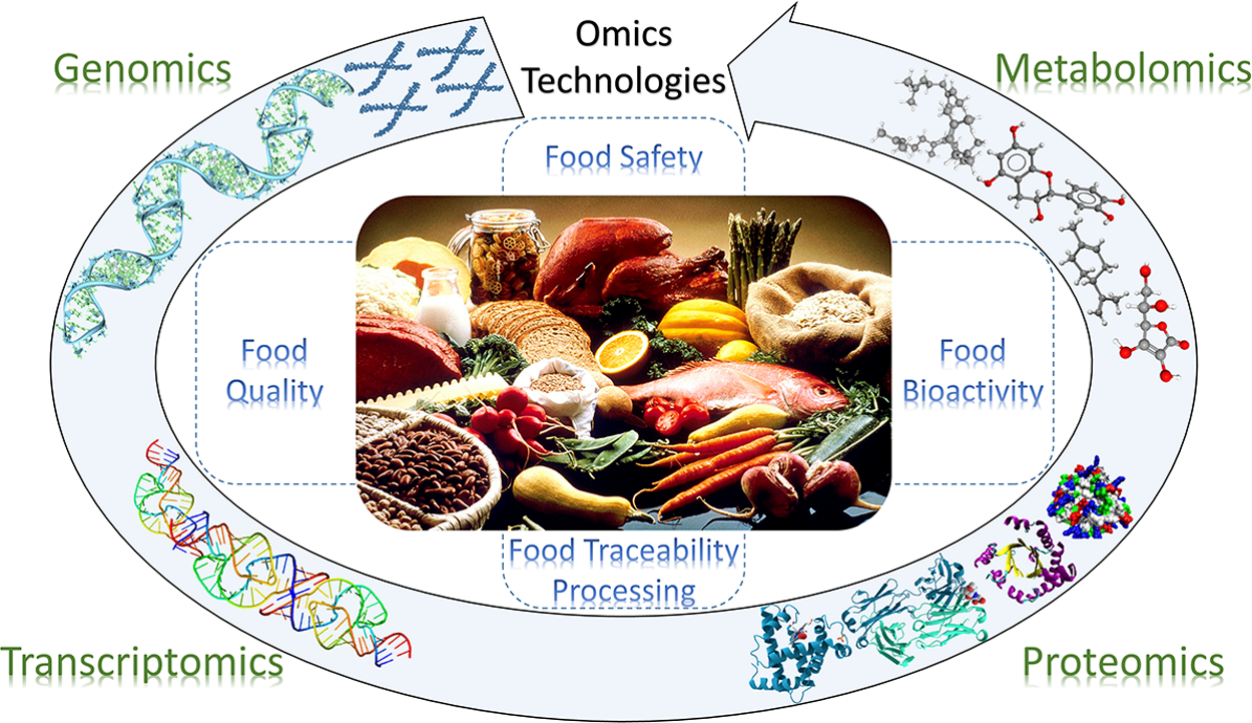
Schematic representation of the omics technologies and areas of food science covered by Foodomics.

As already stated, Foodomics involves the use of multiple omics tools (genomics, transcriptomics, proteomics, and metabolomics) to provide molecular information on the different expression levels (i.e., gene, transcript, protein, or metabolite), and to integrate this information from a systems biology perspective (Figure 1). The first step when performing any Foodomics analysis is the study design and sample selection, and different materials such as biological fluids (blood, plasma, urine, saliva, or cerebrospinal fluid) or solids (tissue, cells, feces), as well as food-derived products in its drinkable (milk, yogurt, wine) or solid (meat, seafood, cereals) forms, can be used.

In human nutrition, genomics (the comprehensive analysis of DNA structure and function) has been used to study how diet may affect the expression of genetic information, and how an individual’s genetic makeup affects the metabolism and response to nutrients and other bioactive components in food;^35^ but genomics can also be used to determine species present in a food sample, reveal the names, types, and proportions of microorganisms, and track foodborne disease agents.^36^ In contrast with the genome, which is characterized by its stability, the transcriptome (or the complete set of RNA molecules expressed in one organism at a specific time) is dynamic, because it changes in response to a wide range of factors. There are three main techniques to investigate the transcriptome, with the fundamental goal of identifying differentially expressed genes (DEGs) between the conditions studied: real-time quantitative polymerase chain reaction (RT-qPCR), gene expression microarrays, and next-generation RNA sequencing (RNA-Seq) techniques.^37,38^ Each of these techniques possesses different advantages and drawbacks. On the one hand, RT-PCR is characterized by its high sensitivity and specificity, and relatively low cost, but it requires the design of specific primers for each target gene and it only allows for the analysis of a limited number of genes at the same time. On the other hand, gene expression microarray has been the standard technology in transcriptomic studies, because it allows the analysis of thousands of transcripts simultaneously; however, prior knowledge of the target genome for probe design is needed and quantification accuracy is limited by background noise and fluorescence saturation. Finally, RNA-Seq technology has revolutionized many fields of biology and has emerged as an attractive alternative to RT-qPCR and gene expression microarrays, as it enables the full sequencing of the entire transcriptome and the detection of RNA sequence variants and isoforms. RNA-Seq technology has rapidly evolved recently, improving the quality and yield of the available platforms, and reducing their costs, but there are still some limitations, such as the time required for sequencing and the complex and extensive data analysis.^39,40^ In the food science field, RNA-Seq technology has been applied, for instance, to sequence the major crops to study the genetic diversity and for improving crop adaptations,^41^ or for the identification and quantification of microbial organisms,^42^ among other applications. Together with the technological advances, these transcriptomics techniques require advanced bioinformatics tools to produce meaningful data. In the case of microarray data analysis, these tools allow one to read and check the raw data, but also perform the normalization, filtering, and selection steps prior to identifying DEG.^43,44^ In the case of RNA-Seq, bioinformatics are also needed for data quality control, read alignment, de novo assembly, or transcript discovery, and for quantification, validation, and visualization of the results.^45^

Genomics and transcriptomics techniques are complemented by proteomics, which represents the comprehensive scientific study of all expressed proteins or entire proteome at any given time in an organism. The proteome is a dynamic reflection of genes/transcripts and the environment, and it has been used for biomarker discovery in clinical diagnosis, to study the effects of nutrients on human protein expression, the changes in food under certain conditions, or for the identification and validation of bioactive food peptides and their health effects.^46^ A variety of proteomic approaches for reliable quantification of individual proteins and/or food proteome are available, and they have been widely applied in food research, quality control, authenticity assessment, safety control, and food bioactivity.^47^ The expansion of proteomics in the food science field has been possible, because of the application of high-resolving separation systems (mainly liquid chromatography (LC)), together with high sensitivity and high-resolution (HR) tandem mass spectrometry (MS). However, and despite these advances, proteomes are very complex and their study must face several challenges, such as a large dynamic range of concentrations and biochemical properties (charge, size, or hydrophobicity), protein modifications (phosphorylation, acetylation, or glycosylation), sophisticated protein conformational changes, and protein–protein interactions, which makes difficult to get a complete view of the proteome in a single analysis. Because of this variability, several protein extractions methods have been developed and one or more purification and/or separation steps are usually required upfront for MS analyses.^48^ These separation steps can be performed at the intact protein level (known as the “top-down” approach) or at the peptide level after protein digestion (known as the “bottom-up” approach), and they are typically based on gel electrophoresis and/or LC.^49^ After separation, the analysis of the isolated proteins or peptides is based on MS detection, using soft ionization methods such as matrix-assisted laser desorption/ionization (MALDI) and electrospray ionization (ESI). Finally, protein identification is based on the comparison of the experimental data with data stored in databases (such as GenBank, RefSeq, UniProt, UniRef, or EMBL-EBI).^50^ These databases are continuously growing and contain information about protein amino acid sequence, post-transcriptional modifications, protein localization, and MS/MS spectral information for many different species. This information can be downloaded and combined with bioinformatics tools that allows the systematic analysis of the high-throughput data obtained, where Sequest, Mascot, Andromeda, X!Tandem, and PEAKS DB are some of the most-used search engines.^51^ However, when experimental data cannot be matched with data contained in databases, the experimental MS/MS spectra must be interpreted de novo, manually, or using specialized software.^52^

Finally, metabolites (or the end products of cellular regulatory processes) represent the downstream products of multiple interactions between genes, transcripts, and proteins. The metabolome includes a huge variety of endogenous and exogenous classes of compounds (such as amino acids, fatty acids, carbohydrates, vitamins, and lipids) with differences in size, polarity, and compound concentration. In addition, the metabolome is constantly changing, because of all of the chemical reactions occurring in the studied system (blood, plasma, cell, tissue, or food). In order to assess the biochemical diversity, sample preparation is a critical step in metabolomics, because it varies, depending on the analytical method and the type of metabolite to be analyzed. Because of the broad physicochemical diversity of the metabolome and the wide concentration range of the metabolites in the biological samples, several extraction methods have been developed, mainly based on the polarity of the metabolites, and not a single method can extract the full metabolome.^53,54^ Apart from the sample preparation, different separation techniques such as LC, gas chromatography (GC), or capillary electrophoresis (CE), as well as different detection techniques, such as MS and nuclear magnetic resonance (NMR), can be used for sample analysis. For instance, GC-MS technique is ideal for identifying and quantifying small acids, alcohols, hydroxyl acids, amino acids, sugars, fatty acids, sterols, catecholamines, drugs, and toxins. However, some compounds cannot be analyzed by GC-MS, and other analytical methods based on LC and CE have been developed for this aim.^53,54^ In addition to the separation technique, many other variables (such as mobile phase, stationary phase, pH, or ionic strength) can be selected for the specific analysis of a group of metabolites. For instance, reverse phase (RP) stationary phase is frequently used in LC analyses to separate nonpolar metabolites (such as nonpolar vitamins, sterols or triacylglycerols), while hydrophilic interaction liquid chromatography (HILIC) is preferred for the study of very polar metabolites (such as amino acids, sugars, or acylcarnitines). Another important parameter to be selected when analyzing metabolites is the ionization mode. Electron ionization (EI) is usually selected for analyses of small, nonpolar, and volatile organic compounds (VOC); and soft ionization techniques, including ESI and atmospheric pressure chemical ionization (APCI) are chosen to ionize thermally labile and moderately polar organic analytes.^55^ The combination of all these extraction, separation, and detection techniques generates complex data matrices that requires the use of advanced bioinformatics tools and processing strategies to extract biologically relevant information about metabolites.^56,57^ Several open-source bioinformatics tools, such as XCMS,^58^ MZmine,^59^ xMSanalyzer,^60^ OpenMS,^61^ or MS-DIAL^62^ have been developed for data processing including peak detection, deconvolution, and alignment, noise filtering and normalization, among other steps. In addition, univariate and multivariate statistical analysis using unsupervised models such as principal component analysis (PCA), cluster analysis (HCA), and nonlinear mapping (NLM), and supervised models such as linear discriminant analysis (LDA), partial least discriminant analysis (PLS-DA) or orthogonal partial least discriminant analysis (OPLS-DA) are commonly performed.^56,63^ Metabolite identification is then performed by calculating the molecular formula (based on the exact mass and isotopic pattern obtained from HR-MS instruments), and by comparing the experimental MS/MS spectra against EI mass spectral fragmentation or MS/MS fragmentation databases.^64^ Actually, the most important metabolomics databases are METLIN,^65^ the Human Metabolome Database (HMBD),^66^ the Mass Bank of North America (MoNA) (https://mona.fiehnlab.ucdavis.edu/), NIST (https://chemdata.nist.gov/), mzCloud (https://www.mzcloud.org/), and the Global Natural Product Social Molecular Networking (GNPS),^67^ which have been continuously growing during the past decade, both in coverage and chemical diversity. However, confident metabolite identification is still a bottleneck in the metabolomics process, and new approaches for elucidating or predicting the structures of novel metabolites are being developed, mainly based on advanced computational algorithms and quantum chemistry.^68,69^

But integrating and interpreting the enormous amount of data generated by the above-mentioned omics platforms also require the development of bioinformatics tools to get a holistic view from a Foodomics perspective. Many tools are available in order to reduce the complexity of the data and to build and visualize genes, proteins, and metabolites and their interaction networks, according to regularly updated databases.^70−73^

## Foodomics Applications

### Foodomics for Food Safety

Food industry globalization has turned into a reality nowadays, favoring the production, distribution, and food trade around the world. However, this fact also involves the increase of food pollution associated with different environmental and anthropogenic agents that should be carefully investigated and controlled. This fact has brought about the development of numerous regulations and guidelines published by different renowned institutions to control food safety. In addition, it is also increasing the concern of consumers about the relevance of food products on health and, consequently, their interest in knowing and understanding information about diet and food products. Hence, great efforts must be done in order to ensure the safety of consumers and guarantee the quality of food, improving current regulations, searching for new control strategies and good quality markers, and providing adequate information to the general population.^22,74^

The development of efficient approaches that allow a reliable assessment of hazardous substances or components that can endanger the safety and quality of food has been an important challenge recently within the area of analytical chemistry. In this sense, the analysis of toxic chemicals, dangerous pathogens, or objectionable materials in food products have constituted the main action lines. Considerable improvements have been already done, in terms of sensitivity, selectivity, reproducibility as well as simplicity, cost reduction, and sustainability in this way. It is expected that the evolution of targeted and untargeted approaches shall allow the suitable determination of a higher number of compounds in a short time, which is one of the main objectives in this field.^10,32^

Foodomics is a recognized discipline to increase and ensure the standards of food safety, in which the application of high-throughput technologies such as genomics, transcriptomics, proteomics, and metabolomics constitutes the main tool to achieve such objectives.^75^

As it is well-known, the evaluation of pathogens is one essential action line to ensure food safety, as well as to avoid the generation of food waste.^76^ Apart from that, the evaluation of chemical contaminants has also a great importance, since there are numerous toxic substances commonly used in industry, agricultural, or medicine, among other areas, that can appear in food constituting an important risk for consumers, because of their toxicity, which increases with the development of a wide range of diseases.^10,77^

A thorough revision of the most recent literature regarding the application of Foodomics in food safety shows that most of the applications developed in the last two years have been focused on the evaluation or the study of the influence of pathogens in food matrices. In this regard, different types of studies have been performed intended for the evaluation of the effect of specific pathogens on the spoilage of food matrices,^78,79^ as well as the study of relevant markers during product storage, under certain conditions.^76,80^ In those studies, most of them based on qualitative analysis, NMR, or LC techniques hyphenated to MS were used for analytes determination.

For instance, the spoilage of food matrices was evaluated by Lou et al. in fish matrices, including fish sticks and broths.^78^ Considering that the main cause of fish putrefaction is microbial activity, authors evaluated the modifications in the metabolic profiles when sterile fish sticks and broths were inoculated with *Shewanella baltica* strains, based on the results of previous studies in which such specie was isolated from spoiled fish. After 10 days of storage at 4 °C, metabolites were extracted using a mixture of methanol/water (1/2; v/v) three times and the extracts were lyophilized prior to dilution in deuterated water and subsequent analysis by NMR. In both matrices the formation of toxic biogenic amines from amino acids was observed, as well as inosine and hypoxanthine, from the degradation of adenine nucleotides, which are involved in the development of autoimmune disorders.

**Figure 2:**
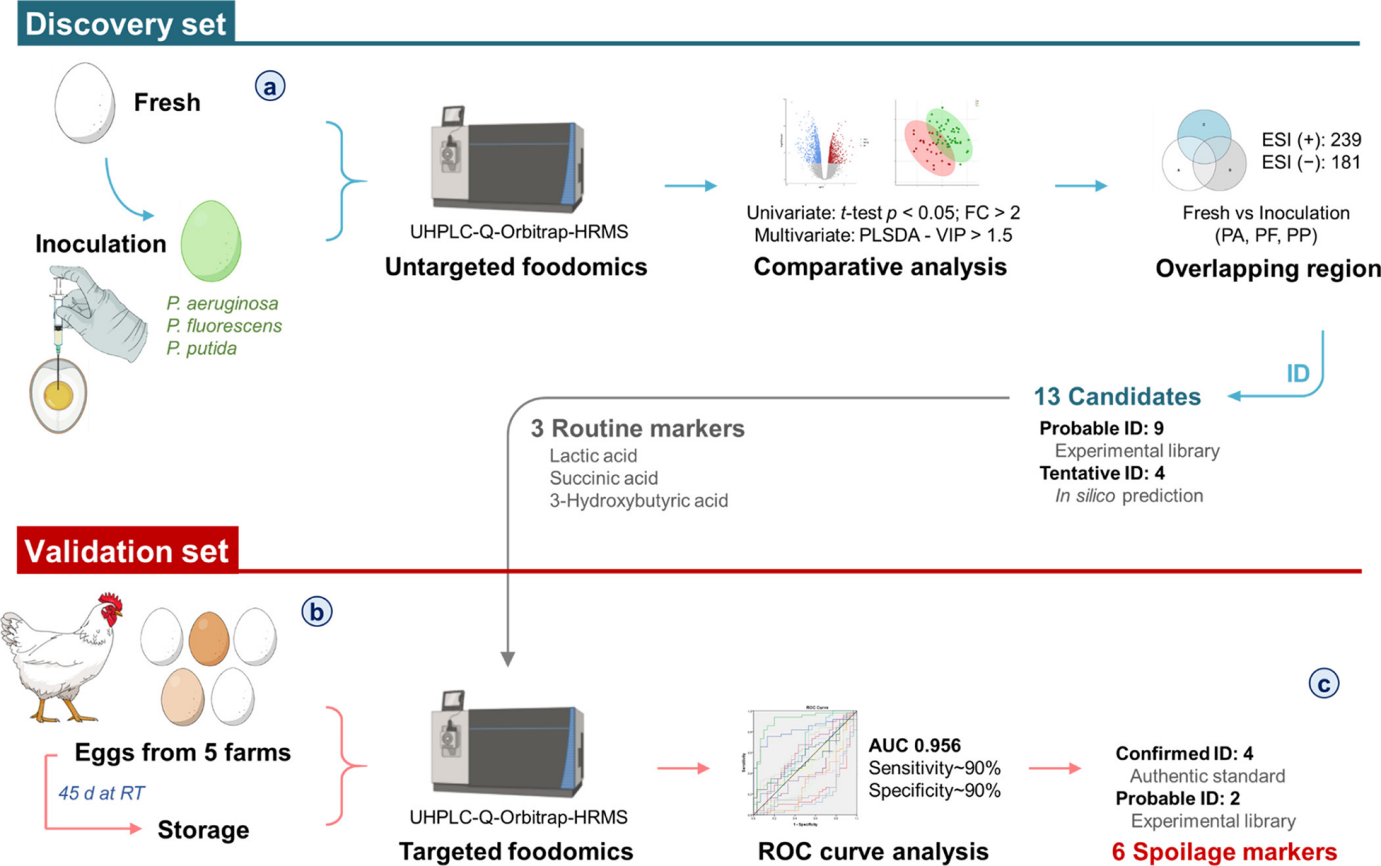
Complete workflow of the foodomics strategy, including inoculations and analytical steps (i.e., extraction, UHPLC-MS/MS analysis and statistical studies), for the evaluation of novel markers in chicken egg spoilage after treatment with three *Pseudomonas* bacteria commonly present in this type of matrices. [Reprinted with permission from ref (79). Copyright 2021, American Chemical Society, Washington, DC.]

In the same way, Chang et al. performed a thorough assessment of novel markers in spoiled chicken eggs using an UHPLC-Q-Orbitrap-MS/MS instrument.^79^ In this case, both targeted and untargeted strategies were applied after the extraction of the metabolites from eggs using ultrasound-assisted extraction with methanol as solvent. Markers were annotated by spectral matching with authentic standards, experimental libraries, or in-silico fragmentation. Targeted metabolomics was employed to verify the markers in eggs from five different farms, as shown in Figure 2, in which the entire process, including the inoculation with three bacteria species (i.e., *Pseudomonas aeruginosa*, *P. fluorescens*, and *P. putida*) and the analytical strategy are schematically represented. Results revealed an increase in lactic and 3-hydroxybutyric acids and decreases in four phospholipids, including phosphocholine, lysophosphatidylethanolamine (LPE(O-18:1)), and lysophosphatidylcholine (LPC(16:0) and LPC(18:0)), which were highlighted as new markers. These data remark the necessity of including more regulatory analyses in eggs after separation from their shells and mixing to ensure hygienic quality and freshness of that product.

These two studies demonstrate the relevance of Foodomics as a suitable tool to further understand the degradation processes of food product by the wide evaluation of metabolomic profiles, which provided valuable information that can be used to establish more-accurate mechanism to prevent food degradation, decreasing the generation of food waste and ensuring consumer wellbeing.

Apart from studies in food spoilage, omics techniques have been also applied for the safety evaluation of food during storage. In this sense, Wu et al. evaluated the possible inactivation effect against *Listeria monocytogenes* on salmon, using electrolyzed water combined with moderate heat treatment by a metabolomics approach using NMR determination, after metabolites extraction using a mixture of phosphate buffer saline and acetonitrile and cold sonication.^80^ Forty-three (43) metabolites were characterized, and their detailed study demonstrated that the combination of both antibacterial treatments provided better bacteria inactivation (55%) than electrolyzed water (35%) or heating (25%), separately.

Particularly remarkable is the study performed by Bellassi et al., in which the effect of *Pseudomonas fluorescens* grown under cold chain conditions was evaluated using a combination of metabolomics and proteomics studies.^76^ In that case, two different sample pretreatments of cold storage milk previously inoculated with the strains, were performed for metabolomic analyses. For this purpose, application of liquid–liquid extraction using methanol at 3% (v/v) in formic acid or dichloromethane at 3% (v/v) in formic acid was applied. After that, UHPLC-Q-TOF-MS analysis working in full scan mode was performed, and an untargeted metabolomic approach was used with a level 2 of accuracy with putative identification. For proteomics; filtration, incubation in dithiothreitol (56 °C, 40 min), and final incubation in iodoacetamide (room temperature, 40 min) for alkylation were applied prior to analysis by nano-LC-Q-TOF-MS/MS. Metabolomic data related the presence of phosphatidylglycerophosphates and glycerophospholipids to the level of contamination and allowed detecting lipid and protein degradation products that were directly correlated with the degradative metabolism of *P. fluorescens*. Regarding the results obtained from proteomic study, those corroborated the proteolytic propensity of *P. fluorescens*-contaminated milk, although with lower sensitivity than the metabolic strategy. Therefore, peptide profiles seem to be an adequate complementary technique to metabolomics for the evaluation of strain contamination only when microbial growth is abundant.

Although to a lesser extent, the use of Foodomics has been applied to the evaluation of chemical contaminants. Recently, von Eyken et al. developed an untargeted metabolomics screening of plastic migrants in honey samples, commercialized in both glass and plastic jars, by HPLC-Q-TOF-MS/MS after a simple sample preparation approach constituted by dilution of the pure matrix with a mixture of acetonitrile and water, filtration, and further dilution with water until a 1% honey extract is obtained.^81^ Data analysis allowed the identification of 662 putative potential plastic migrants and two of them, 2-ethyhexyladipate (DEHA) and tris (2-butoxyethyl) phosphate (TBOEP), were confirmed and quantified by using analytical standards. Note that, for comparison of chemical burden between samples sold in glass or plastic recipients, different approaches were applied, finding different conclusions. Unique entity analysis with 100% detection did not find any relevant compounds, and a volcano plot with *p* < 0.05 found just two compounds. However, a data treatment approach based on the differential frequency of detection found 13 compounds in glass and 40 in plastic, 6 of which were unique to honey samples sold in plastic jars and 3 were unique to honey samples sold in glass jars. The varied results suggest that the relatively low frequency of contaminants in food must be taken into account for comparing groups. This fact highlights the relevance of selecting the appropriate data treatment approach in these types of studies, in which a huge amount of information is obtained, which represents a powerful tool that should be adequately used to obtain reliable information and solid conclusions, especially in those cases where consumer health is compromised by food contamination.

### Foodomics for Food Quality

Consumers frequently judge food quality based on multiple aspects related to food appearance, origin, food composition, taste, flavor, or food nutritional properties. With a growing consumer demand for food quality, there is a clear need for the development of novel analytical methods to meet the highest quality standards. The analytical methodologies available for food quality validation are commonly based on the use of biomarkers and profiling techniques for the characterization of food matrices and identification of adulterants.^82^ In this context, Foodomics tools like metabolomics have found great applicability in characterizing and establishing similarities and differences among food products; providing essential information to understand sensorial and nutritional food properties, as well as to determine the food fingerprint as the signature of food quality and authenticity.

**Figure 3:**
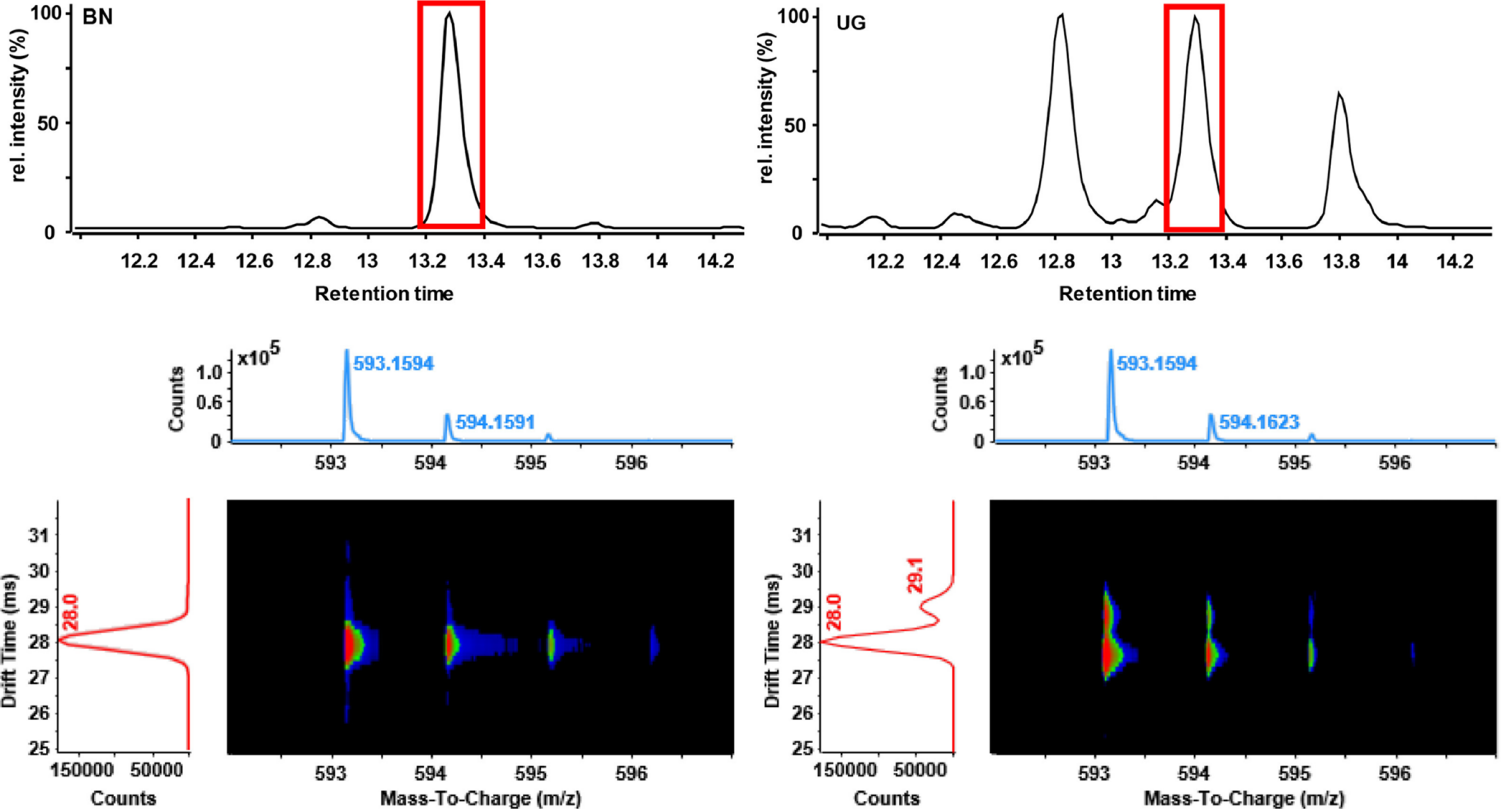
LC-IM-QTOF-MS separation. Top panel shows extracted ion chromatogram (*m*/*z* 593.1594) of the BN sample (left) and the UG sample (right). Bottom panel shows mass and drift spectrum of the highlighted peak for the BN sample (left) and of the UG sample (right) showing a clear separation of an isobaric compound only present in the UG sample. [Reprinted with permission from ref (83). Copyright 2020, Elsevier.]

To distinguish beverages with different qualities and origins, advanced analytical methods with high separation capacity are required to resolve the variety and number of compounds; some of them with multiple isomeric forms that are difficult to be separated by conventional chromatographic approaches.^83^ Hence, comprehensive chromatographic methods based on two-dimensional gas chromatography (GC × GC) were recently reported to monitor the volatile profile or fingerprint of cachaça liquors,^84^ and beer samples.^85,86^ Traditional approaches using GC × GC-MS are typically based on pixel- or peak-table data processing, which is frequently more demanding, in terms of computational resources, requires an experienced analyst, and is also time-consuming. Ferreira et al. developed a GC×GC-MS methodology for the authentication of cachaça samples, using a column set comprised of a primary Trace TR-5MS column and a secondary HP-50 column.^84^ Detected VOCs were processed by making use of the images generated from the 2D chromatograms for multivariate data processing, and applying DD-SIMCA as a simple one-class classifier method. Volatile metabolites of lager beer were also studied in an in-depth profiling analysis combining green head space solid-phase microextraction (HS-SPME) with GC×GC-TOF-MS.^85^ The orthogonal separation achieved using an Equity-5 column (^1^D) and a DB-FFAP column (^2^D) coupled to the TOF analyzer increased the chromatographic and spectral resolution and also the sensitivity, allowing the simultaneous analysis of 329 volatile compounds (major and trace analytes) that can be further used in beer quality control or monitoring brewing steps. A similar methodological approach was proposed by Paiva et al. to assign the contribution of Brazilian Ale 02 yeast strain to the aroma profile of beer.^86^ A DVB/CAR/PDMS fiber was used for HS-SPME extraction of VOCs that were subsequently analyzed by FM-GC×GC-MS. Since chromatograms generated by GC×GC are structurally complex and contain a lot of information, a multiway principal components analysis (MPCA) was selected to extract all meaningful information, using pixel-based pattern recognition. These works highlight the importance of combining high-peak-capacity techniques, with appropriate data processing techniques. Montero et al. reported an alternative multidimensional strategy based on LC hyphenated to ion mobility spectrometry coupled to high-resolution mass spectrometry (LC-IM-Q-TOF-MS) to characterize the main phenolic compounds in eight different herbal liqueurs.^83^ This approach provides high sensitivity and great peak capacity, mainly due to the three separation dimensions; that is, liquid chromatographic (compounds polarity), ion mobility (shape-to-charge ratio), and time-of-flight mass spectroscopy (TOF-MS), as exemplified in Figure 3.

New quality classification models based on metabolite patterns or fingerprints to discriminate samples of different origin, different animal origin, or with different organoleptic attributes have been recently investigated. Thus, an olive oil quality classification model was developed and validated after GC-MS analysis with dynamic headspace entrainment, followed by thermal desorption (DHS-TD).^87^ Chromatographic data mining was performed using the recently developed PARADISe software for peak deconvolution, which has been useful for tentative identification of unknown compounds, when matching their spectra with NIST libraries.

Other discrimination methods were also reported to prevent fraud activities in the dairy and fish industries, using UHPLC-Q-Orbitrap-MS analysis and chemometric tools. Thus, Jia et al. proposed an untargeted MS-metabolomics approach, monitoring under data-independent acquisition (DIA) mode to obtain spectra for all precursor ions in order to facilitate the comprehensive identification of unknown compounds.^88^ Differences in the molecule profiles of raw milk from different animal species (cow milk, goat milk, and water buffalo milk) were observed for discriminant analysis. β-Carotene was found only in cow milk; ergocalciferol was found only in water buffalo milk; and the contents of nonanoic acid, decanoic acid, and octanoic acid were higher in goat milk than those in cow milk and water buffalo milk. On the other hand, Chang et al. investigated potential indicators for fish freshness operating under data-dependent acquisition (DDA) mode, following a three-stage Foodomics workflow involving the filtering and selection of metabolites, identification of metabolite structures by spectrum mapping and further verified through time-dependent analysis.^89^ The loss of freshness in fish is manifested in an increase of metabolites involved in nucleotide changes (uracil, hypoxanthine, and inosine), lipid hydrolysis (α-linolenic acid, docosahexaenoic acid, arachidonic acid, and linoleic acid) and a decrease in decanoylcarnitine involved in fatty acid metabolism.

While metabolomics often focuses on more water-soluble compounds, lipidomics studies can also provide relevant information on the lipid-rich fraction of foods, being a complementary omics tool to evaluate food quality. Thus, an untargeted lipidomics strategy was proposed by Sutliff et al. to assess the molecular composition of a lipid-rich fraction of bell peppers that can be related to color.^90^ The results of the analysis by HPLC-Q-TOF-MS, followed by statistical analysis using linear mixed effects regression and false discovery rate, suggested that the compound most strongly associated with color was the carotenoid β-cryptoxanthin. Another LC-Q-TOF-MS-based lipidomics survey compared the lipid composition of human milk (HM) and formula milk (FM), targeting different lactation stages and infant age range.^91^ Nutritionally important lipids, such as long-chain polyunsaturated fatty acids containing lipid species, sphingomyelines, or ether analogues of glycerophosphoethanoloamines were detected in HM collected in all studied lactation stages, when compared to FM.

Despite the restricted sensitivity of NMR-based methods, the combination of highly reproducible, noninvasive, rapid, and simple-use proton nuclear magnetic resonance (^1^H-NMR) with multivariate statistical analysis in Foodomics applications has emerged over the last decades for the implementation of models to trace the food quality.^92^ Along this line, a combination of NMR spectroscopy and chemometrics was proposed by Cavallini et al. to characterize beer.^93^ The authors compared two multivariate approaches: the full spectra analysis and the analysis of the chemical features extracted by multivariate curve resolution. In addition, PCA was used for exploratory purposes; pareto scaling was used to preprocess the NMR spectra dataset, while autoscaling was used to preprocess the features dataset. The resolved information is comparable with the full spectrum information, but interpretability is greatly improved. Another NMR-based Foodomics approach was proposed by Herbert-Pucheta et al. to discriminate between wine samples produced from the same Cabernet Sauvignon variety fermented with different yeast strains.^92^ A double pulsed field-gradient-echo (DPFGE) NMR methodology was applied in this work as a selective refocusing method of the aromatic frequency (5.5–10 ppm) of the wine samples fermented, which allowed one to discriminate between yeast strains used for the controls and for large-scale alcohol reductions after supervised standard and sparse PLS-DA multivariate statistical treatments.

The huge potential of Foodomics tools has also been implemented in meat quality control. Proteomic analysis was shown to be a high-throughput approach for identification of peptide biomarkers in meat samples. For instance, to predict pale soft exudative-like defects in cooked hams, Théron et al. developed a predictive method to classify raw material prior to ham processing using blood samples, by studying the spectral data of a proteomics analysis of plasma.^94^ Spectral fingerprints of proteins and their secondary structures were obtained using MALDI-TOF-MS and Attenuated Total Reflectance - Fourier Transform Infrared (ATR-FTIR) spectroscopy, respectively. Another proteomics approach was proposed by Zhu et al., who were looking for biomarkers of tenderness on Irish cattle, based on nano-LC-Q-Orbitrap-MS analysis.^95^ The bioinformatics analysis make use of proteomic resources such as the *Bos taurus* database for LC-MS/MS raw files aligned, ProteINSIDE and STRING web service databases for Gene Ontology analyses, and evaluation of protein–protein interactions, respectively.

Other Foodomics applications combining metabolomics, metagenomics and statistical tools have been addressed to study the quality of raw milk for the production of hard cheese.^96^ Discriminant milk metabolites specific of each feeding conditions were identified by UHPLC-Q-TOF-MS metabolomics analysis, followed by supervised multivariate statistics. The metagenomic profile of *Staphylococcaceae*, *Pseudomonadaceae*, and *Dermabacteraceae* was found to be significantly correlated to discriminant milk metabolites.

### Foodomics for Food Traceability and Processing

Food traceability is an essential issue of the Foodomics domain that provides precise information on food origin and food composition throughout all stages of the food supply chain, through primary production, processing, distribution, and retailing. These monitoring processes from farm production to consumer, which are very often defined as “from farm to fork”,^97^ have a strong influence on food quality. In this regard, new Foodomics approaches using metabolomics, proteomics, and genomic tools have been recently reported to provide precise and reliable traceability systems in order to warranty food quality and safety. Thus, the knowledge of the processing method, distribution process, composition, and geographical origin of the end-food product are the main motivations in food traceability studies, as discussed below.

Recent applications have demonstrated the potential of metabolomics approaches to evaluate food traceability and investigate molecular changes during food processing. Thus, analytical methods based on HR-MS instruments; mainly using hybrid Q-TOF-MS or Q-Orbitrap-MS analyzers, hyphenated mainly to UHPLC, are the most widely reported. The most used chromatographic separations are based on C18 columns for metabolomics applications, whereas HILIC stationary phases are the option of choice for lipidomics. Mobile phases composed of water and acetonitrile with different modifiers (i.e., 0.1% formic acid, ammonium formate) are frequently used. ESI or heated electrospray ionization (HESI) sources are the most popular and widespread used interfaces in HR-MS based metabolomics coupled to LC techniques, operating in positive ESI mode; although some approaches are reported to operate in both positive and negative ESI modes to obtain complementary structural information.^98^

HR-MS-based metabolomics has been proposed to investigate qualitative traits of meat, allowing the simultaneously detection of a wide range of metabolites related to processing, ripening, and shelf life conditions of meat products. For instance, the molecular processes promoted by the addition of three different microbial starters (i.e., *Pediococcus pentosaceus*, *Staphylococcus xylosus*, and *Lactobacillus sakei*) during the manufacturing of dry-fermented salami was investigated by Rocchetti et al.^99^ The untargeted UHPLC-Q-Orbitrap-MS analysis revealed that each microbial starter imposed distinctive metabolomic signatures at the end of ripening, involving lipids (including hydroxy and epoxy derivatives of fatty acids) and γ-glutamyl peptides that contribute to the final sensorial quality of products. Rocchetti et al. also performed an untargeted screening of dry fermented sausage metabolites by UHPLC-Q-TOF-MS.^100^ Fermented sausages produced following a cold drying-ripening process at the lower relative humidity values (65%–80%) showed several oxidation markers at the end of ripening, such as oxy and hydroxy derivatives of fatty acids. In the first study, the collected raw data obtained from Orbitrap were converted into .abf format and further processed using the software MS-DIAL, and annotated via spectral matching against the MoNA database.^99^ In the latest study, the raw mass features from Q-TOF were processed in the software Profinder (Agilent Technologies), based on the targeted “find-by-formula” algorithm, and the identification of meat metabolites was achieved against the comprehensive database FoodDB.^100^

The molecular mechanisms of the fermentation processes in commercial beverages and condiments were investigated using metabolomic and also lipidomics approaches. UHPLC-Q-TOF-MS/MS untargeted metabolomics allowed investigation of the bioaccessibility of health-related metabolites from oat beverages at the intestinal level.^101^ The annotations from tandem MS/MS data were processed using MS-DIAL software, the publicly available MS/MS experimental spectra built into the software MoNA, and MS-Finder in-silico fragmentation from compounds in the FoodDB. Under this approach, a broad range of flavonoids, phenolic acids (avenanthramides), amino acids, and steroids were identified (17 compounds using MS/MS annotation workflow, and 184 metabolites putatively annotated with FoodDB). Jia et al. studied the dynamic changes during Fu brick tea fermentation applying an untargeted profiling strategy, involving an untargeted screening mode (i.e., variable-data-independent acquisition, vDIA) and the combination of C18 and a HILIC columns for metabolomics and lipidomics analysis, respectively.^102^ Using a single C18 column, Li et al. analyzed the chemical profile of Pixian doubanjiang during fermentation, operating in ESI(±)-Q-TOF-MS modes to broaden the range of detectable compounds.^98^ A total of 99 differential metabolites were obtained, including amino acids, small peptides, fatty acids and lipids, sugars, organic acids, biogenic amines, and nucleosides). Kyoto Encyclopedia of Genes and Genomes (KEGG) (https://www.kegg.jp/) online tools were used to evaluate the effect of the fermentation process on its metabolic pathways.

The effect of storage on the metabolomic profile of biochemically active immature plants like microgreens (i.e., red beet and amaranth) was evaluated by an untargeted metabolomics profiling analysis.^103^ Using a custom database built by combining annotations from Phenol-Explorer and Food Database, 316 compounds were identified at the level 2 of accuracy (i.e., putatively annotated compounds), consisting of mainly polyphenols and lipids. The phenolic content values were found to be significantly higher after 10 days of storage. Cooking is another important food processing stage that can transform some food compounds by oxidations, and/or thermal degradations. Lozano-Castellón et al. comparatively assessed the effect of different cooking methods on the phytochemical profile of extra-virgin olive oil (EVOO), considering both its hydrophilic and lipophilic fractions.^104^ The phenolic profiling was performed by UHPLC-ESI (+)-Q-TOF-MS, whereas lipidomic profiling was obtained by UHPLC-ESI(±)-Q-Orbitrap-MS. Conventional cooking methods (oven, pan frying, and deep frying) produced more oxidation products (epoxy and hydroxy derivatives of lipids) and markedly induced degradation processes, compared to new vacuum cooking techniques.

The combined use of GC-MS and LC-MS platforms has been recently implemented to investigate liquor aging processes, as well as processing methods affecting peanut oil components. Thus, the molecular mechanism of the role of a special storage container (Mare Nectaris) in the aging process of Feng-flavor of Baijiu liquor was unveiled through Foodomics analysis.^105^ UHPLC coupled to Q-Orbitrap-MS allowed the accurate identification of most small molecules, especially nonvolatile components, whereas volatile metabolites such as esters and other aroma components were analyzed by GC-MS. Classification of Feng-flavor Baijiu, considering the aging category, was also performed with the proposed Foodomics approach.^106^ The complementary information provided by both chromatographic platforms in MS-based metabolomics allow one to study how processing methods affect peanut oil composition and nutrition in rats.^107^ Fingerprinting analysis of serum and liver samples, using a HP-5MSI column for GC and a Hypersil GOLD C18 for LC, revealed more than 50 different biomarkers, including amino acids, lipids, carbohydrates, and nucleoside compounds. The metabolic pathway analysis revealed that hot-pressed and hydroenzymatic peanut oil can ameliorate hepatic metabolic disorders caused by a high-fat diet.

Although the geographical origin of beef is most commonly determined using genomics approaches, stable isotope ratio analysis, and multielemental analysis, an untargeted metabolomics approach, including both UPLC-Orbitrap-MS and GC-MS, was proposed by Man et al.^108^ Using a UPLC HSS T3 column for and a UPLC and HP-5MS column for GC analysis, the chemical profiles obtained operating in positive and negative ESI modes for UPLC-Orbitrap-MS analysis and GC-MS showed potential biomarkers for beef from different countries, including amino acids, several sugar metabolites, and a number of phosphatidylcholines and phosphatidylethanolamines.

**Figure 4:**
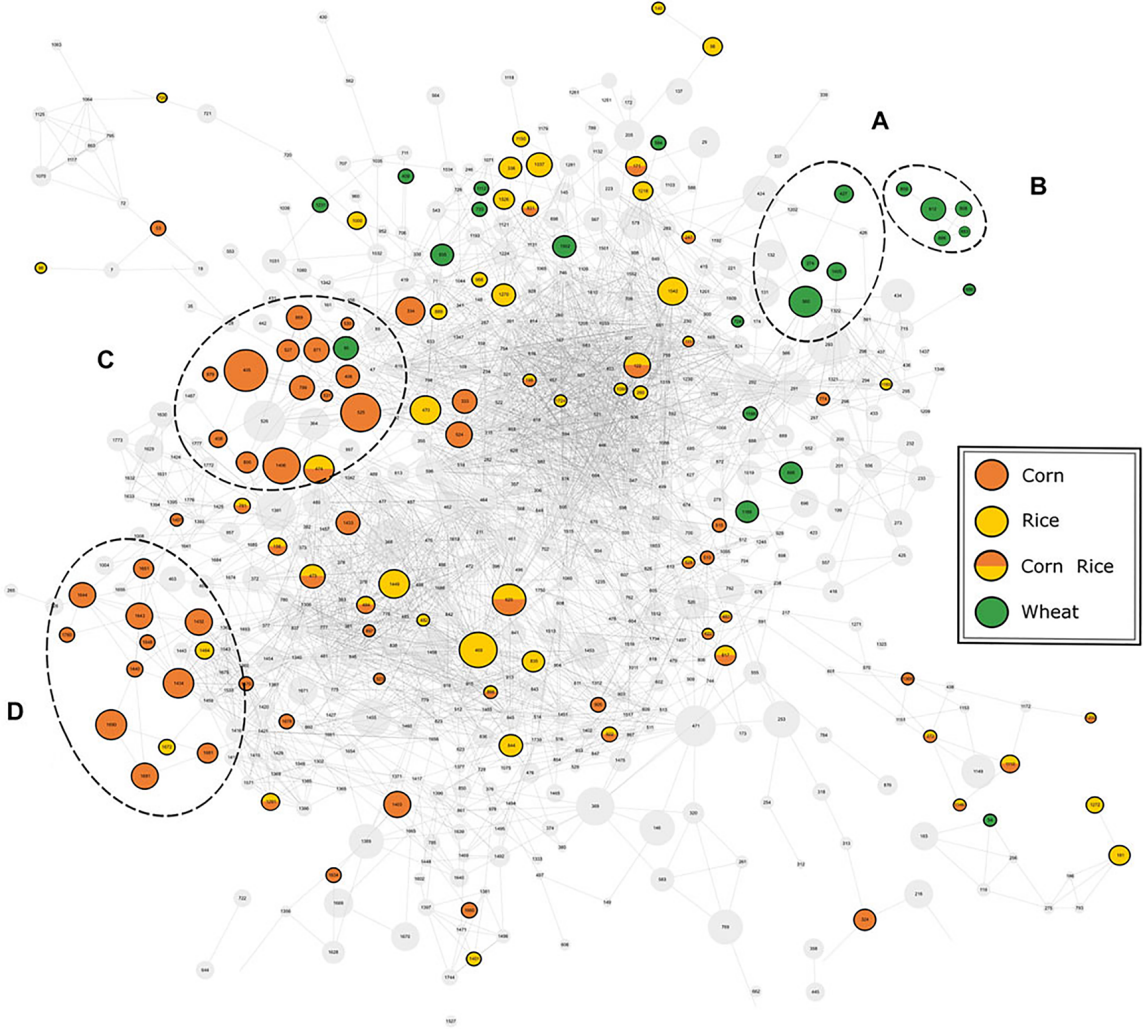
Mass spectral similarity network of the fragmentation spectra of compounds detected by UPLC-TFF-MS. The nodes representing the respective compounds are connected by edges representing their spectral similarity. Compounds found to be specific for a carbohydrate source are colored accordingly. Two cluster of potential markers are highlighted for (A, B) wheat and (C, D) corn. [Reprinted from ref (110). Copyright 2021, Frontiers, Lausanne, Switzerland.]

Other works have also demonstrated the power of ultra-HR-MS approaches based on flow-injection Fourier transform ion cyclotron mass spectroscopy (FI-FT-ICR-MS) to provide a comprehensive picture of the beer’s metabolome by assigning thousands of unambiguous molecular formulas to the mass signals.^109^ The study of exact mass differences through different visualization methods (i.e., Van Krevelen diagrams, PCA and OPLS-DA scores, and loading plots) was proposed as a valuable tool to monitor the formation of Maillard reaction products and to better understand their chemical interplay. In another work, the authors also analyzed the influence of different starch sources (barley, wheat, corn, and rice) on the metabolic signature in the final beer product, by both DI-FT-ICR MS and UPLC-TOF-MS (Figure 4).^110^ Reversed-phase UPLC-TOF-MS was used to access information about molecular structures (MS2-fragmentation spectra) and isomeric separation, with a focus on less-polar compounds. This enabled a deeper characterization through exact mass values and fragmentation mass spectra.

To a lesser extent than HR-MS technology, ^1^H NMR-based metabolomics has emerged recently as a valuable technology to trace the origin, manufacture, or authenticity of food products.^111^ Despite its relatively low sensitivity, this technology is highly reproducible, rapid, and no preliminary sample separation is required, and it has been implemented to screen the metabolic profile of the blue mussel (*Mytilus edulis*) and the Manila clam (*Ruditapes phlippinarum*),^112^ to discriminate the geographical origins of agave species and grape varieties,^113^ to determine the metabolomic profiling of acerola clones at different ripening stages,^114^ to evaluate the rheological characteristics of sponge cake after in vitro digestion,^115^ or to investigate milk fermentation during yoghurt production when different heat treatments of milk and starter cultures are employed.^116^ In all of these studies, advanced statistical tools played a critical role for compound identification and the discovery of significant metabolites. For instance, the statistical total correlation spectroscopy (STOCSY) was used by Aru et al. to identify mytilitol as an integral part of the metabolome of *R. philippinarum* and *M. edulis*, but also to suggest that the distribution of this metabolite is species-specific and dependent on the geographical origin of the sample.^112^ In the case of acerola investigations, multivariate regression modeling was essential to predict the concentrations of ascorbic acid and total phenolic content for each ripening stage of the different acerola clones.^114^ And the PCA and OPLS-DA results obtained by Huang et al. showed that the addition of *Eucheuma* as a fiber-rich flour replacer in sponge cake reduced the release of amino acids and fatty acids during in vitro digestion, and a mathematical model was developed to describe the glucose release results quantitatively.^115^

Proteins are also essential macronutrients of foods known to confer technological and organoleptic properties,^117^ and proteomics studies can be used to investigate the molecular changes occurring during food processing and for food authentication purposes.^47,118^ In this context, a recent proteomics study was focused on the evaluation of the effects of maturation time and simulated gastrointestinal digestion on the molecular and peptide profiles of “Bresaola Valtellina” meat product, by using SDS-PAGE, size exclusion HPLC, HPLC-LTQ-MS, 2DE-MALDI-TOF-MS and ^1^H NMR.^119^ This study demonstrates that meat ripening makes proteins more bioaccessible, and the release of peptides smaller than 250 Da could be responsible for the inhibitory activity on amylolytic enzymes and for the antioxidant properties exhibited by the digests. Another group of interesting proteins widely studied in the food science field are gluten proteins. These proteins contribute to the rheological properties of dough from cereals such as wheat, barley and rye, but they can also cause food intolerance and allergies.^120^ In a recent study, the characterization of gluten proteins in wheat flours of different technological qualities was performed by nanoUPLC-Q-TOF-MS^E^, showing different proteomics patterns between the samples, and identifying low molecular weight glutenin subunits as upregulated proteins in superior-quality wheat flours.^121^

Finally, metagenomics based on NGS technologies has been widely used to improve food safety and food quality, because it allows for the characterization of the diversity of microbial communities and their ecological interactions within food.^122^ In addition, these technologies can also be used to monitor the microbial composition and their interactions during food storage conditions. An example of this approach is the use of qPCR and high-throughput 16S rRNA gene sequencing (16S rRNA-seq) to study the stability of microbial composition in drinkable yogurt during shelf life.^123^ The authors evaluated yogurts produced with traditional yogurt starter cultures including *Bifidobacteria* or without them, showing that formulations with the starter cultures including *Streptococcus* and *Lactobacillus* were associated with a lower abundance of each probiotic, compared to those that additionally had *Bifidobacterium* in the starter culture.

### Foodomics for Food Bioactivity

Another major goal of Foodomics is the investigation of bioactive compounds, which are defined as nonessential constituents that typically occur in small quantities in foods that can modulate one or more metabolic processes, resulting in the promotion of better health conditions. These compounds widely vary in chemical structure, and they can mainly be grouped as polyphenols, phytosterols, terpenoids, polysaccharides, carotenoids and tocopherols, glucosinolates, triterpenes, alkaloids, capsaicinoids, bioactive peptides, and polyunsaturated fatty acids.^124^ Furthermore, these compounds can provide with health benefits by diverse molecular mechanisms, and Foodomics can help to understand these processes, but also to investigate the presence, bioavailability, and biological characteristics (such as toxicity, antioxidant, antiproliferative, or anti-inflammatory properties) of these interesting molecules in different food matrices.

Generally, the investigation of the molecular mechanisms involved in the beneficial properties of bioactive compounds is a complex task, because of the multiple interactions that can occur between these components and the biological systems; therefore, a systems biology approach is desired. This approach is characterized by the use of different omics technologies (i.e., genomics/transcriptomics, proteomics, and metabolomics), but integrating these omics platforms remains an ongoing challenge for many researchers. In this regard, only a few works have addressed the integration of multiomics approaches, and standalone metabolomics technology has been the most used in the last two years. In addition to the aforementioned limitation, the use of complex biological systems, such as humans, makes it difficult to interpret the results obtained, and therefore less complex in vitro and in vivo models are frequently used. These models offer several advantages, such as the reduction of the duration and costs, and the identification of possible associated risks.

**Figure 5:**
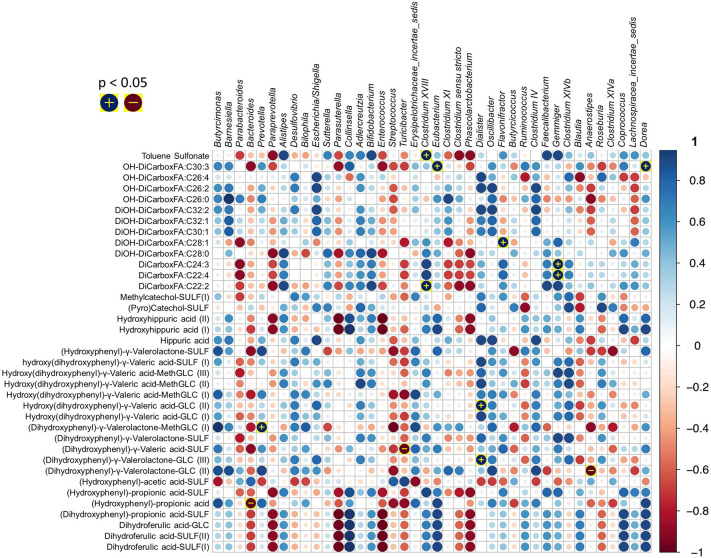
Heatmaps correlating area under curve (apple polyphenol extract) of metabolites measured over 5 h in blood and genus level 16S rRNA relative abundance of faecal microbiota present in each subject. [Reprinted with permission from ref (127). Copyright 2018, Elsevier.]

Following this research line, a cell culture in vitro model of human colorectal cancer (HT-29 cells) was selected to evaluate the antiproliferative capacity of two bioactive extracts from different food matrices: *Passiflora mollissima* seeds^125^ and *Physalis peruviana* L. calyx^126^). In these works, the molecular changes in cells after the different treatments were evaluated using gene expression microarrays (for transcriptomics) and UHPLC-Q-TOF MS/MS (for metabolomics) analyses. In the case of *P. mollissima* seeds, the resulting extract was enriched in polyphenols (flavonoids, genuine flavan-3-ols, and proanthocyanidins oligomers), which markedly affected the viability of HT-29 colon cancer cells, whereas minor effects were observed on normal human colon fibroblast cells.^125^ The use of a Foodomics approach revealed that more than 500 genes were differentially expressed and 22 metabolites were altered, some of them involved in the polyamine and glutathione metabolism, and the alteration of the intracellular ceramide levels. In the case of *P. peruviana* calyx extract, the main constituents were withanolides, phenolic acids, flavonoids, sucrose esters, terpenoids, phytosterols, and phytol derivatives; and this extract also affected the viability of HT-29 cells, blocking the cells in the S phase of the cell cycle.^126^ Moreover, more than 3200 genes and 24 metabolites were significantly altered, many of them involved in glutathione redox reactions, in the pyrimidine ribonucleotide interconversion, or in the carnitine shuttle and β-oxidation of fatty acids processes. In a more complex work involving human subjects, metabolomics (using a HPLC-Orbitrap MS/MS instrument) was applied to investigate the metabolic products of various classes of apple polyphenols upon ingestion, and to describe the nutrikinetics of these metabolites in plasma and urine samples.^127^ In addition, fecal samples were collected from each individual during the study for 16S rRNA gene profiling. Authors identified a large number of microbial catabolites (valerolactones and valeric acids) of apple flavanols (catechins and procyanidins), and the presence of methylcatechol metabolites, vanillactic, vanilpyruvic, and homovanilic acid, suggesting a possible impact of apple polyphenols on catecholamine metabolism. Moreover, significantly positive correlations were found in plasma and urine between valeric acid, valerolactone and (epi)catechin metabolites and *Dialister*, *Prevotella* and *Escherichia* bacterial genus, while the presence of these compounds were negatively associated with *Anaerostipes*, *Turicibacter*, *Lachnospiracea incertae sedis*, *Coprococcus* and *Blautia* (Figure 5).

Apart from these studies, standalone metabolomics technologies have been also used to compare the phenotypes between two conditions after a specific treatment with bioactive compounds or different diets. For instance, a comprehensive lipidomics study (based on UHPLC-Q-TOF MS/MS) was performed to study the neuroprotective and anti-inflammatory potential of an olive leaves extract enriched in triterpenoid compounds using an in vitro model of Alzheimer’s disease.^128^ The authors of this work demonstrated that the secretion of three cytokines related to inflammation (interleukin-6, interleukin 1β, and tumor necrosis factor-α) were decreased after the treatment, and this extract also protected SH-SY5Y neuroblastome cells against the toxic effect of amyloid β-peptide. In addition, more than 250 intracellular lipids were identified, with a great number of phosphatidylcholines and phosphatidylethanolamines significantly increased, whereas several triacylglycerols were decreased, suggesting triterpenoids from olive leaves as good neuroprotective candidates. In another study, ^1^H NMR has been used to investigate the alteration of the human urine metabolome after the consumption of two different diets: the New Nordic Diet (NND) and the Average Danish Diet (ADD).^129^ The NND was higher in fish, whole grain, fruit, and vegetables, and lower in meat than the ADD. By analyzing the metabolome of 142 centrally obese Danes (20–66 years old), the authors of this work identified different effects related to the diet, season, sex, and changes in body weight, reflecting changes in protein and carbohydrate metabolism between the two diets.

A wider group of metabolomics studies have been focused on the investigation of the presence, bioavailability, and biological characteristics of bioactive compounds, being polyphenols the most targeted ones. For instance, the research group of Hassine et al. performed a comprehensive analysis of the phytochemical profile on seeds from three *Lupinus* species, including one cultivar (*Lupinus albus*) and two wild accessions (*Lupinus cossentinii* and *Lupinus luteus*), collected from the northern region of Tunisia.^130^ The untargeted metabolomic profiling using an UHPLC-Q-TOF MS/MS instrument allowed the identification of 249 compounds, with a great abundance of phenolics and alkaloids. The identification of these compounds was performed by using Profinder B.06 (from Agilent Technologies) and MS-DIAL softwares, and by using the publicly available MS/MS MoNA database and MS-Finder in-silico fragmentation from compounds reported in FoodDB and PlantCyc databases. Among the three different species, *L. cossentinii* was the most abundant source of polyphenols (mainly tyrosol), followed by *L. luteus* and *L. albus*. In addition, *L. cossentinii* also had the highest reductive power (based on CUPRAC assay), but *L. albus* had the highest radical scavenging capacity (based on ABTS assay). In another work, the same LC-MS instrumentation and metabolite identification workflow was used to investigate the polyphenolic profile of leaves, stems and roots from *Cydonia oblonga*.^131^ Several compounds were identified in the different parts of the plants, including flavonoids (i.e., anthocyanins, flavones, flavan-3-ols, and flavonols), phenolic acids, low-molecular-weight phenolics (tyrosol equivalents), lignans, and stilbenes. Based on different in vitro assays (DPPH, ABTS, FRAP, and CUPRAC), leaves showed the highest antioxidant potential, stems showed the highest acetyl- and butyryl-cholinesterases inhibitory capacity, and fruit were the only parts inhibiting the α-glucosidase enzyme. Lastly, the chemical profile of different rosemary cell lines has been assessed by the combination of two complementary analytical technologies (UHPLC-Q-TOF MS/MS and GC-Q-TOF MS).^132^ A total of 71 compounds, including hydroxycinnamic acid and hydroxybenzoic acid derivatives, flavonoids, phenolic diterpenes and triterpenes, unsaturated fatty acids and their esters, phytosterols, and carotenoids were identified in the rosemary extracts. In addition, the antiproliferative potential against human HT-29 colorectal cancer cell line was evaluated, revealing that the viability of HT-29 colon cancer cells was mostly affected after treatment with a white rosemary extract.

The bioavailability and bioaccessibility of food bioactive compounds are also important aspects to be investigated by Foodomics.^133^ In the case of bioaccessibility, in vitro gastrointestinal digestion models have been the most widely used to study several food matrices. This is the case of EVOO, which were subjected to an in vitro gastrointestinal digestion and the changes in bioactive compounds were evaluated following an untargeted metabolomics approach based on UHPLC-Q-TOF MS/MS analyses.^134^ This methodology allowed the identification of 219 sterols and 67 polyphenols in EVOO samples, and demonstrated that raw EVOO samples were richer in total sterols and tyrosol than digested samples. More specifically, flavonoids were the most affected compounds after in vitro digestion, while relatively high bioaccessibility values were obtained for tyrosol equivalents. Of special interest was the conversion of oleuropein–aglycone (i.e., the major phenolic compound in EVOO) to hydroxytyrosol, increasing more than 7 times the values of the latter compound after digestion. Following a similar methodology but including a fermentation step simulating a large intestine process, the bioaccessibility of different phenolic compounds from nonedible pomegranate parts,^135^ or from blackberry puree polyphenols after dietary fiber addition,^136^ were monitored by the same research group. In the case of pomegranate, the most abundant compounds in undigested extracts were polyphenols, terpenoids, sterols, alkaloids and amino acids, which showed a higher abundance in leaves.^135^ In addition, the in vitro digestion results indicated a wide transformation of polyphenols after 24 h of digestion, mainly for phenolic acids and tyrosols in flowers (probably because of the insoluble dietary fiber content). In the case of blackberry puree polyphenol, the untargeted profiling evidenced that the free phenolic fraction of blackberry puree was characterized mainly by flavonoids, phenolic acids, lignans, and other low-molecular-weight polyphenols, showing clear differences from the bound phenolic fraction detected.^136^ Authors also observed that the interaction between phenolics and soluble dietary fiber decreased the total phenolic content, the total antioxidant capacity and the monomeric anthocyanin content of blackberry samples. However, increased levels of soluble dietary fiber modulated the bioaccessibility of phenolics, which also promoted the formation of low-molecular-weight compounds such as 4-vinylphenol, benzoic acid, tyrosol, and other phenolic acids. In a different work, the in vitro bioaccessibility investigation of artichoke constituents was complemented with a bioavailability study by using an intestinal cell culture in vitro model (Caco-2 cells).^137^ In this work, authors detected a large abundance of phenolic acids and sesquiterpene lactones in raw material, but a decrease in polyphenols and sesquiterpene lactones content was observed after 20 h of in vitro large intestine fermentation. The highest bioaccessibility values were obtained for flavonoids such as anthocyanin and flavone equivalents, and relatively high bioavailability values were obtained for flavonols, phenolic acids, and sesquiterpene lactones. Other techniques, such as HR-NMR, have been used to investigate the effect of balsamic vinegar dressing (BVD) on the digestibility and component release of cheese, cured meat, and boiled potatoes.^138^ BVM modulated the protein digestion of cheese and cured meat by inhibiting pepsin in the gastric phase, while it reduced the release of total carbohydrates in boiled potatoes, which was consistent with a reduction of the pancreatic amylase activity. Finally, the effect of the in vitro gastrointestinal digestion (including a final step with purified brush border membrane enzyme preparations) on the peptidome of hemp flour and hemp protein isolates was evaluated by 2-DE-LC-ESI-Q-Orbitrap MS.^139^ The results of this work demonstrated that only a limited number of peptides could survive the digestion process, highlighting that none of them came from hemp allergens. Conversely, some released peptides contained amino acidic motives that could be associated with their bioactivity. Taken together, the results of the presented works highlight the important role of bioavailability and bioaccessibility aspects on the potential beneficial properties of bioactive compounds.

## Conclusions and Foreseen Foodomics Challenges

The development of advanced analytical tools and their application through a Foodomics perspective have opened new possibilities to expand the knowledge on the food science field. This Review summarized the main advances made in the food safety, food quality, food traceability and processing, and food bioactivity subfields, highlighting the important role of transcriptomics, proteomics, and metabolomics, together with biostatistics, chemometrics, and bioinformatics tools. However, omics approaches are still underused in this field, because of expensive instrumentation and the high level of experience and technical skills needed for method development, as well as for software management and statistical data analysis. Furthermore, to understand the impact of diet on health as a whole, it is necessary to consider many parameters, just to mention a few: the broad nature of food molecules, the microbiota, the interindividual variability, the food dynamic processing starting from the ingestion, and followed by the digestion in the gastrointestinal tract, the intestinal transference to the circulation, the transformation by the liver, the usage by every organ, and the final excretion in urine and feces.

In the transcriptomics field, the RNA-seq technology is becoming more affordable and has been applied to the characterization of transcriptomes of different foods, and its wider application in the study of the effects of bioactive food compounds is expected. Other tools, such as molecular engineering of microorganisms through clustered regularly interspaced short palindromic repeats (CRISPR)-Cas9, together with synthetic biology applications pose a great potential to modify microbial communities in food, improving processes such as fermentation or generating enhanced probiotic strains. In the proteomics field, the combination of more sensitive, faster, and higher-resolution MS instruments coupled to different separations systems and fractionation techniques will increase the coverage of proteomes, subproteomes, and peptidomes. However, there are still some limitations when the time aspect is considered, which is essential to understand the metabolic and physiological changes occurring during molecular and cellular processes. In the case of metabolomics, great advances in extraction, separation, and detection techniques have been performed (such as the introduction of ion mobility analysis), but the main limitations are still the identification and accurate quantification of metabolites. Another major challenge is the integration of the different omics approaches, because of the lack of adequate bioinformatics tools and our limited understanding of the biological and chemical process occurring inside any biological system, what makes especially demanding the study about the effect of food components on health. The achievement of all these goals also requires of a collaborative work within the scientific community to compare and share data. Therefore, more harmonized and standardized sampling methods, improvements in computational techniques and biological databases (i.e., with functional annotations), and further developments in the analytical technologies used on each specific omics field are essential.

Overcoming the above-mentioned limitations will allow scientists to gain a more comprehensive Foodomics insight about the relationship between food and health, while reinforcing the control of food safety, quality, traceability, and processing.
